# Spontaneous Hair Cell Regeneration Is Prevented by Increased Notch Signaling in Supporting Cells

**DOI:** 10.3389/fncel.2018.00120

**Published:** 2018-05-04

**Authors:** Melissa M. McGovern, Luyi Zhou, Michelle R. Randle, Brandon C. Cox

**Affiliations:** ^1^Department of Pharmacology, School of Medicine, Southern Illinois University, Springfield, IL, United States; ^2^Department of Surgery, Division of Otolaryngology, School of Medicine, Southern Illinois University, Springfield, IL, United States

**Keywords:** hair cell regeneration, cochlea, Notch, CreER, Tet-On, *NICD*

## Abstract

During embryonic development, differentiation of cochlear progenitor cells into hair cells (HCs) or supporting cells (SCs) is partially controlled through Notch signaling. Many studies have shown that inhibition of Notch signaling allows SCs to convert into HCs in both normal and drug damaged neonatal mouse cochleae. This mechanism is also implicated during HC regeneration in non-mammalian vertebrates; however, the mechanism of spontaneous HC regeneration in the neonatal mouse cochlea is less understood. While inhibition of Notch signaling can force SCs to convert into HCs and increase the number of regenerated HCs, it is currently unknown whether this pathway is involved in spontaneous HC regeneration observed *in vivo*. Therefore, we investigated the role of Notch signaling during the spontaneous HC regeneration process using *Atoh1-CreER^TM^::Rosa26^loxP-stop-loxP-DTA/^*^+^ mice injected with tamoxifen at postnatal day (P) 0 and P1 to ablate HCs and stimulate spontaneous HC regeneration. Expression changes of genes in the Notch pathway were measured using immunostaining and *in situ* hybridization, with most changes observed in the apical one-third of the cochlea where the majority of HC regeneration occurs. Expression of the Notch target genes *Hes1, Hes5, Hey1, HeyL*, and *Jagged1* were decreased. To investigate whether reduction of Notch signaling is involved in the spontaneous HC regeneration process, we overexpressed the *Notch1 intracellular fragment (N1ICD)* in cochlear SCs and other non-sensory epithelial cells in the context of HC damage. Specifically, *Atoh1-CreER^TM^::Rosa26^loxP-stop-loxP-DTA/^*^+^*::Sox10^rtTA^::TetO-LacZ::TetO-N1ICD* mice were injected with tamoxifen at P0/P1 to stimulate spontaneous HC regeneration and given doxycycline from P0–P7 to induce expression of *N1ICD* as well as *LacZ* for fate-mapping. We observed a 92% reduction in the number of fate-mapped regenerated HCs in mice with *N1ICD* overexpression compared to controls with HC damage but no manipulation of Notch signaling. Therefore, we conclude that increased Notch signaling prevents spontaneous HC regeneration from occurring in the neonatal mouse cochlea. Understanding which components of the Notch pathway regulates regenerative plasticity in the neonatal mouse cochlea will inform investigations focused on stimulating HC regeneration in mature cochlea and eventually in humans to treat hearing loss.

## Introduction

Hearing loss, one of the most prevalent conditions in the world, can result from multiple etiologies such as aging, viral infection, or exposure to noise or ototoxic drugs. In those born with normal hearing, death of sound-sensing hair cells (HCs) located within the cochlea is the most common source of hearing impairment. The mature mammalian cochlea does not regenerate HCs after damage, thus hearing loss is permanent (Bohne, 1976; Hawkins et al., 1976; Oesterle et al., 2008). However, in non-mammalian vertebrates, including birds, fish, and amphibians, the inner ear responds to damage by regenerating lost HCs throughout the life of the animal (reviewed in Bermingham-McDonogh and Rubel, 2003; Stone and Cotanche, 2007). Several studies have suggested that the Notch signaling pathway is involved in the HC regeneration process in non-mammals (reviewed in Stone and Cotanche, 2007; Brignull et al., 2009).

The Notch pathway uses membrane bound ligands and receptors expressed on adjacent cells to generate a mosaic pattern of cell types. In the mammalian cochlea, HCs and neighboring supporting cells (SCs) develop from the same pool of progenitor cells through a Notch-mediated process. As progenitor cells take a HC fate, they begin to express the Notch ligands Delta-like ligand 1 (Dll1), Delta-like ligand 3 (Dll3), and Jagged2 (Jag2) (Lanford et al., 1999, 2000; Morrison et al., 1999; Kiernan et al., 2005; Brooker et al., 2006; Hartman et al., 2007). These ligands signal to neighboring progenitor cells through Notch receptors which inhibits them from becoming HCs and instructs a SC fate (Lanford et al., 2000; Zheng et al., 2000; Zine et al., 2001; Murata et al., 2006; Hayashi et al., 2008; Doetzlhofer et al., 2009). A recent study reported limited down-regulation of Notch target genes after HC damage in the neonatal mouse cochlea (Korrapati et al., 2013). However, inhibition of the Notch signaling pathway, using pharmacological agents or genetically modified mouse models, in the late embryonic and early postnatal cochlea has the consistent effect of inducing the conversion of SCs into HCs either in the undamaged cochlea (Yamamoto et al., 2006; Doetzlhofer et al., 2009; Li et al., 2015; Maass et al., 2015) or after HC loss (Korrapati et al., 2013; Mizutari et al., 2013; Bramhall et al., 2014; Hu et al., 2016). Therefore, active Notch signaling in SCs plays an important role in maintaining their cell fate in the neonatal cochlea.

Recently, several groups have demonstrated that the immature murine cochlea has the capacity to spontaneously regenerate HCs during the first postnatal week (Bramhall et al., 2014; Cox et al., 2014; Hu et al., 2016) and loss of Notch signaling has been shown to increase the degree of HC regeneration over that which occurs spontaneously in HC damaged cochlear explants (Korrapati et al., 2013; Bramhall et al., 2014; Hu et al., 2016). While these results demonstrate that SCs can be forced to convert into HCs through inhibition of the Notch pathway, it is unclear whether the Notch signaling pathway is involved in the mechanism of spontaneous HC regeneration. To address this question, we first investigated changes in Notch signaling during the spontaneous HC regeneration process in the neonatal mouse cochlea and found that the expression of the Notch target genes *Hes1*, *Hes5, Hey1, HeyL*, and *Jagged1* (*Jag1*) were decreased. Next, to determine whether the spontaneous HC regeneration process is inhibited by sustained Notch signaling, we combined HC damage with overexpression of the *Notch1 intracellular domain* (*N1ICD*) in SCs and other non-sensory epithelial cells and measured the effect on spontaneous HC regeneration. This model showed a 92% reduction in the number of spontaneously regenerated HCs and suggests that weakened Notch signaling contributes to SC plasticity which underlies spontaneous HC regeneration.

## Materials and Methods

### Animals

*Plp-CreER^T2^* mice (Jax stock #5975, Doerflinger et al., 2003), *Rosa26^loxP-stop-loxP-DTA^* mice (Jax stock #6331, Ivanova et al., 2005), *Rosa26^loxP-stop-loxP-tdTomato^* mice (also called Ai14, Jax stock #7914, Madisen et al., 2010), and *TetO-LacZ* mice (Jax stock #2621, Furth et al., 1994), were obtained from The Jackson Laboratory (Bar Harbor, ME). *Atoh1-CreER^TM^* mice (Chow et al., 2006) were provided by Dr. Suzanne Baker (St. Jude Children’s Hospital, Memphis, TN, United States); *Hes5^LacZ^* mice (Imayoshi et al., 2010) were provided by Dr. Ryoichiro Kageyama (Kyoto University, Kyoto, Japan); *Sox10^rtTA^* mice (Ludwig et al., 2004; Walters and Zuo, 2015) were provided by Dr. Michael Wegner (Erlangen University, Erlangen, Germany); and *TetO-N1ICD* mice (Stanger et al., 2005) were provided by Dr. Ben Stranger (University of Pennsylvania, Philadelphia, PA, United States). Genotyping for all mouse lines was performed by Transnetyx, Inc. (Cordova, TN, United States). Mice of both genders were used in all studies and all animal work was performed in accordance with approved animal protocols from the Institutional Animal Care and Use Committee at Southern Illinois University School of Medicine.

### Substances Given to Animals

To induce CreER recombination for either HC death or tdTomato expression, tamoxifen (Sigma-Aldrich, St. Louis, MO, United States) was dissolved in 100% corn oil and injected intraperitoneally (IP) at 3 mg/40 g on either postnatal day (P) 0, or both P0 and P1. To induce *LacZ* and *N1ICD* overexpression in cochlear SCs and other non-sensory epithelial cells, doxycycline (100 mg/kg, IP, Fisher, Hampton, NH, United States) was injected at P1 (∼6 h after the second tamoxifen injection) as well as administered in the food (2000 mg/kg, Envigo, Huntingdon, United Kingdom) to the nursing mother for the duration of the experiment.

### Real-Time qPCR

Quantification of gene expression was conducted using real time quantitative polymerase chain reaction (qPCR). Neonatal pups were euthanized under isoflurane anesthesia (Henry Schein, Melville, NY, United States) and cochleae were collected from P7 animals, flash frozen in either liquid nitrogen or a dry ice slurry, and then stored at -80°C. Isolation of total RNA was conducted using tri-reagent (Fisher, Hampton, NH, United States), and precipitated with isopropanol (Acros Organics, Geel, Belgium) at -20°C overnight followed by purification with DNAse treatment (cat #AM1906, Fisher, Hampton, NH, United States) and reprecipitated using sodium acetate (Fisher, Hampton, NH, United States). Once total RNA was isolated, the concentration was measured using a Nanodrop 2000 (Fisher, Hampton, NH, United States) followed by reverse transcription into cDNA using Thermo Scientific Maxima First Strand cDNA synthesis kit (cat #K1641, Fisher, Hampton, NH, United States) and stored at -20°C. The expression level of selected genes was measured from cDNA by qPCR using Sybr Green (cat #K0391, Fisher, Hampton, NH, United States) and a CFX Connect Optics Module (Bio-Rad, Hercules, CA, United States). Comparison of gene expression among samples was conducted using the Pfaffl method which is a modified version of the delta-delta *C*t equation that takes into account the efficiency of each primer set (Pfaffl, 2001). Gene expression was normalized to a housekeeping gene (Rpl19), which has been used previously for qPCR experiments in cochlear tissue (Basch et al., 2011; Korrapati et al., 2013; Wan et al., 2014; Maass et al., 2016). Data are expressed as fold change from control. **Table 1** lists the primer sets used for each gene that was investigated and each primer set was tested for specificity with a reverse transcriptase-negative control to ensure they did not detect genomic DNA.

**Table 1:** Primer sets used for qPCR of Notch effector genes.

Gene	Forward primer	Reverse primer	Reference
*Hes1*	TCAACACGACACCGGACAAAC	ATGCCGGGAGCTATCTTTCTT	Chonko et al., 2013
*Hes5*	GCACCAGCCCAACTCCAA	GGCGAAGGCTTTGCTGTGT	White et al., 2006
*Hey1*	CACTGCAGGAGGGAAAGGTTAT	CCCCAAACTCCGATAGTCCAT	Korrapati et al., 2013
*HeyL*	GCGCAGAGGGATCATAGAGAA	TCGCAATTCAGAAAGGCTACTG	Korrapati et al., 2013
*N1ICD*	GACAACTCCTACCTCTGCTTATGCC	TTACTGTTGCACTCGTTGACCTCG	Yamamoto et al., 2006

### Immunostaining

Neonatal pups were euthanized under isoflurane anesthesia and cochleae were collected and post-fixed in 4% paraformaldehyde (Polysciences, Inc., Warrington, PA, United States) for ∼2 h at room temperature (RT). The samples were then transferred to 10 mM Phosphate Buffered Saline (PBS, Sigma, St. Louis, MO, United States) and stored at 4°C. Following whole mount dissection, cochleae were cut into 3 turns of equal length and placed into a 48 well plate for immunostaining as free floating tissue. Samples were blocked and permeabilized at RT for 1 h with a solution of Normal Horse Serum or Normal Goat Serum (10%, NHS, or NGS, Vector Labs, Burlingame, CA, United States), Bovine Serum Albumin (1%, BSA, Fisher, Hampton, NH, United States), and Triton-X-100 (1%, Sigma, St. Louis, MO, United States) in 10 mM PBS prior to the application of the primary antibodies. Primary antibodies were diluted in a solution of 10 mM PBS containing NHS or NGS (5%), BSA (1%), and Triton-X-100 (0.1%) and incubated overnight at 4°C or 37°C. The following primary antibodies were used: chicken anti-β-galactosidase (β-gal, 1:500, cat#ab9361, Abcam, Cambridge, United Kingdom), goat anti-Jag1 (1:500, cat#sc6011, Santa Cruz Biotechnology, Dallas, TX, United States), rabbit anti-myosin VIIa (1:200, cat#25-6790, Proteus Biosciences, Ramona, CA, United States), rabbit anti-S100a1 (1:1000, cat#ab868, Abcam, Cambridge, United Kingdom), and goat anti-Sox2 (1:400, cat#sc-17320, Santa Cruz Biotechnology, Dallas, TX, United States). The next day, samples were washed three times with 10 mM PBS for 5 min each at RT. Alexa fluor-conjugated secondary antibodies (Life Technologies, Waltham, MA, United States) were diluted 1:1000 in a solution of NHS or NGS (5%), BSA (1%), and Triton-X-100 (0.1%) in 10 mM PBS and incubated for ∼3 h in the dark at RT. Following the secondary antibodies, samples were washed three times for 5 min each in 10 mM PBS followed by a 20-min incubation with Hoechst (1:2000, Fisher, Hampton, NH, United States) and three more, 5-min washes in 10 mM PBS at RT. Following immunofluorescent labeling, samples were mounted on slides in Prolong Gold (Fisher, Hampton, NH, United States).

Additionally, chicken anti-β-gal antibodies produced excessive background and therefore to increase the signal to noise ratio, signal enhancer (enough volume to cover each sample, cat#R37107, Thermo Fisher Scientific, Hampton, NH, United States) was applied at RT for 30 min followed by three 5-min PBS washes prior to the blocking/permeabilization step. A low pH antigen unmasking solution (AUM, cat#H-3300, Vector Labs, Burlingame, CA, United States) was beneficial to increase antibody binding to the epitope for the anti-Jag1 antibodies. For this step, samples were incubated in a hybridization oven at 95°C for 45 min with a 1:100 dilution of the AUM solution in ddH_2_O followed by three 5-min washes in 10 mM PBS prior to blocking/permeabilization.

Imaging was conducted using a Leica SP5 or a Zeiss LSM 800 confocal microscope and images were processed with LAS-lite (Leica, Wetzlar, Germany) or Zen Blue lite (Zeiss, Oberkochen, Germany) software.

### *In Situ* Hybridization

Probes were prepared by transfecting plasmids containing the full-length mouse cDNA sequences for *Hes1* (Accession: BC051428, Clone ID: 6478994, Dharmacon, Inc., Lafayette, CO, United States), *Hes5* (Accession: BC103539, Clone ID: 40039948, Dharmacon, Inc., Lafayette, CO, United States), *Hey1* (Accession: BC086635, Clone ID: 6809680, Dharmacon, Inc., Lafayette, CO, United States), and *HeyL* (Accession: BC130263, Clone ID: 40142873, Dharmacon, Inc., Lafayette, CO, United States) into competent *Escherichia coli* cells (Fisher, Hampton, NH, United States) and incubating for 1 h at 37°C before spreading the cells on an agar plate containing the appropriate selection marker and incubating overnight. Once a clone was selected, it was inoculated with the appropriate selection antibiotic and incubated overnight in 250 ml of media containing the selection marker at 37°C, after which cells were centrifuged and the supernatant was decanted. The cells were processed with a ZymoPURE plasmid maxiprep kit (cat#D4202, Zymo Research, Irvine, CA, United States) to isolate the plasmid DNA. Concentration was measured on a Nanodrop 2000 (Fisher, Hampton, NH, United States) and the plasmids were linearized and cleaned up with phenol::chloroform::isoamyl alcohol (Fisher, Hampton, NH, United States). Plasmids were sequenced by GenScript (Piscataway, NJ, United States) and each sequence was entered into BLAST (NCBI, Bethesda, MD, United States) to determine their correspondence to the endogenous mouse mRNA sequence (*Hes1* = 97.4% homology, *Hes5* = 100% homology, *Hey1* = 99.9% homology, and *HeyL* = 99.5% homology). Then the probes were digoxigenin-labeled (DIG; cat# 286 036 910, Roche, Indianapolis, IN, United States) through *in vitro* transcription and cut into ∼300 bp pieces though a mild alkaline bath to facilitate interaction with the endogenous RNA.

Cochleae were fixed in RNase free 4% paraformaldehyde (PFA; Polysciences, Inc., Warrington, PA, United States) for ∼2 h at RT or overnight at 4°C. Following fixation, cochleae were dissected and dehydrated in a graded methanol series (Fisher, Hampton, NH, United States) for 5 min in each concentration [25, 50, 75, and 100% diluted in RNase free 10 mM PBS with 0.1% Tween-20 (PTw) (Sigma, St. Louis, MO, United States)]. Samples were rehydrated through a reverse graded methanol series and transferred to RNase free 2 ml tubes. Samples were washed three times for 5 min each in RNase free PTw at RT. Next, samples were digested for 15 min at RT with 10 μg/ml proteinase K (Fisher, Hampton, NH, United States) in PTw then incubated with 4% PFA containing 0.2% glutaraldehyde (Sigma, St. Louis, MO, United States) for 20 min to halt the digestion. Then samples were again washed twice with RNase free PTw for 5 min each. Next, hybridization buffer [50% formamide (Sigma St. Louis, MO, United States), 1.5× saline-sodium citrate (SSC, Sigma, St. Louis, MO, United States), pH adjusted to 4.5 with citric acid (Sigma, St. Louis, MO, United States), 50 μg/ml yeast tRNA (Sigma, St. Louis MO, United States), 100 μg/ml heparin (Sigma, St. Louis, MO, United States), 0.2% Tween-20 (Sigma, St. Louis, MO, United States), 0.5% CHAPS (3-[(3-Cholamidopropyl)dimethylammonio]-1-propanesulfonate hyd-rate Sigma, St. Louis, MO, United States), and 5mM EDTA pH 8.0, (Millipore, Billerica, MA, United States)] was added to each sample and incubated at 60°C for 3 h followed by the addition of the DIG-labeled riboprobe to the hybridization buffer at a concentration of 0.5 μg/ml (*Hes5*) or 1 μg/ml (*Hes1*, *Hey1*, and *HeyL*) and incubated at 60°C overnight.

The following day, samples were rinsed twice (∼1 min each) with hybridization buffer and washed twice (30 min each) with hybridization buffer at 60°C. Samples were then washed with a 1:1 mixture of hybridization buffer and 1X MABT [maleic acid buffer containing Tween-20, 5X stock concentration of 500 mM maleic acid (Sigma, St. Louis, MO, United States), 744 mM NaCl (Sigma, St. Louis, MO, United States), 44 mM Tween-20 (Sigma, St. Louis, MO, United States) and ddH_2_O and pH adjusted with Tris Base (Roche, Indianapolis, IN, United States) to ∼7.5] for 20 min at 60°C. Samples were then rinsed three times (∼1 min each) with 1X MABT and washed twice (30 min each) with 1X MABT at RT. Samples were blocked with 1X MABT containing 20% sheep serum (Sigma, St. Louis, MO, United States) and 2% Boehringer blocking reagent (Sigma, St. Louis, MO, United States) for 3 h at RT. Next the samples were incubated overnight at 4°C with anti-digoxigenin antibodies (cat#11093274910, Sigma, St. Louis, MO, United States) in 1X MABT at a final concentration of 1:2000.

The following day, samples were rinsed three times (∼1 min each) with 1X MABT and then washed three times (1 h each) with 1X MABT at RT followed by an overnight wash at 4°C. Samples were then washed twice with alkaline phosphatase buffer [100 mM Tris (Sigma, St. Louis, MO, United States) pH 9.5, 50 mM MgCl_2_ (Fisher, Hampton, NH, United States), 100 mM NaCl (Sigma, St. Louis, MO, United States), and 0.1% Tween-20 (Sigma, St. Louis, MO, United States)] for 1 h each and then processed with 4.5 μl/ml nitro-blue tetrazolium (Sigma, St. Louis, MO, United States) and 3.5 μl/ml 5-bromo-4-chloro-3′-indolyphosphate (Sigma, St. Louis, MO, United States) in alkaline phosphatase buffer in the dark between 2 and 20 h. Samples were then washed twice in alkaline phosphatase buffer (5 min each), washed three times in PTw (1 h each), and fixed in 4% PFA for 30 min. Samples were then cleared in 60% glycerol overnight and mounted on slides in 100% glycerol (Fisher, Hampton, NH, United States) and imaged with a Neurolucida system (MicroBrightField, Inc., United States) integrated with a Zeiss AxioImager M2 microscope (Carl Zeiss Microscopy, Germany) or an Olympus IX70 microscope (Olympus Corporation, Waltham, MA, United States). All *in situ* experiments were performed with matched experimental and control samples from the same litter run in parallel.

### Quantification and Statistical Analysis

For quantification of Sox2-positive and Hes5-LacZ-positive cells, images were taken from two representative regions in the apical turn of the cochlea and cells in either a 150 μm or 200 μm region per image were quantified and averaged for each sample. Positive expression of LacZ was determined by the overlap of LacZ labeling and nuclear staining with Hoechst once antibody background was reduced using the imaging software. The same trained examiner counted images for both control and experimental samples to make sure that the same threshold for positive staining was maintained. To quantify the amount of HC regeneration (LacZ-positive HCs) in the model where *N1ICD* was overexpressed, images of the entire cochlea were taken using tile scan with z-series. Each cochlea was measured and divided into six equal sections as previously described (McGovern et al., 2017). For quantification of HCs in cochlea overexpressing *N1ICD*, 200 μm regions were selected from two representative images per cochlear turn and total HCs were quantified and averaged. All data are presented as mean ± standard error of the mean (SEM). Data was analyzed using a one- or two-way ANOVA or a Student’s *t*-test with Graphpad Prism 6.02 (Graphpad Software Inc., La Jolla, CA, United States).

## Results

### Hes5 Expression Is Reduced During Spontaneous HC Regeneration

**FIGURE 1:**
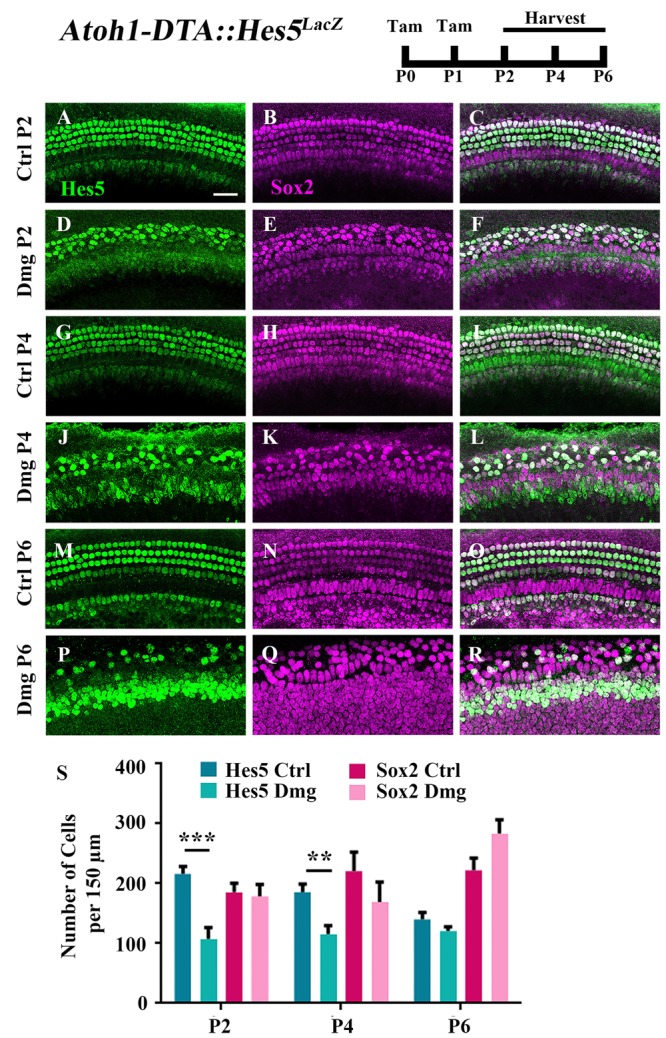
Hes5-LacZ-positive cells, but not Sox2-positive cells, are reduced during the spontaneous HC regeneration process. *Atoh1-DTA::Hes5^LacZ^* mice were injected with tamoxifen (Tam) at P0–P1 to induce HC death and spontaneous HC regeneration and cochleae were collected at P2, P4, and P6. **(A–R)** Representative confocal slice images with anti-β-gal antibodies to detect Hes5-LacZ expression (green) and anti-Sox2 antibodies to label all SC nuclei (magenta). **(A–C**, **G–I, M–O)** Images are taken from P2, P4, or P6 control samples, respectively. Scale bar = 25 μm. **(S)** Fewer Hes5-LacZ-positive cells were detected in HC damaged samples at P2 and P4, but not P6. There was no significant difference in the number of Sox2-positive cells in the *Atoh1-DTA::Hes5^LacZ^* cochlea after HC damage compared to undamaged controls. ^∗∗^*p* < 0.01, ^∗∗∗^*p* < 0.001, as determined by a two-way ANOVA with a Sidak’s *post hoc* test. *N* = 3–5.

**FIGURE 2:**
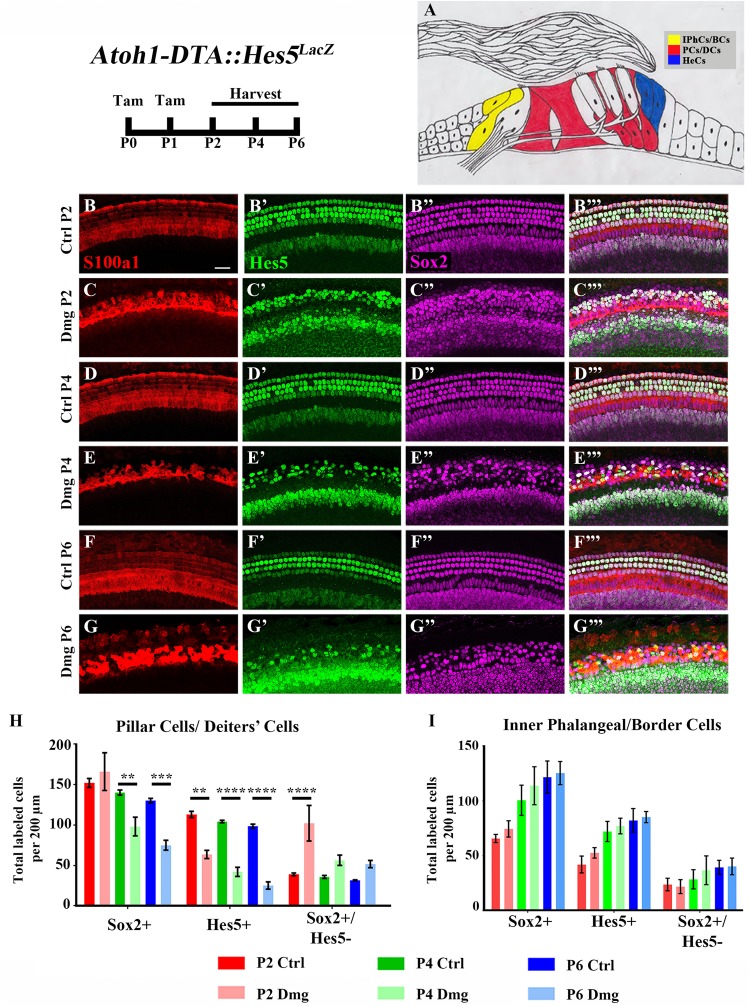
Hes5-LacZ-positive cells are reduced in lateral SCs, but not medial SCs. **(A)** Pillar and Deiters’ cells (red, PCs/DCs) are labeled with S100a1 and inner phalangeal/border cells (yellow, IPhCs/BCs) lie medial to the S100a1 expressing region. Hensen cells (blue, HeCs) express Sox2 and border the lateral edge of the S100a1 region, but do not have Hes5 expression. **(B–G”’)** *Atoh1-DTA::Hes5^LacZ^* mice were injected with tamoxifen (Tam) at P0–P1 to induce HC death and spontaneous HC regeneration and cochleae were collected at P2, P4, or P6. Representative confocal slice images with anti-βgal antibodies to detect Hes5-LacZ expression (green), anti-Sox2 antibodies to label all SC nuclei (magenta), and anti-S100a1 antibodies to label the cytoplasm of PCs/DCs (red). Scale bar = 25 μm **(H)** In the PCs/DCs region, fewer Sox2-positive cells were detected at P4 and P6 after HC damage, while a decreased number of Hes5-LacZ-positive cells were detected at all three ages. Only at P2 were there significantly more Sox2-positive/Hes5-LacZ-negative cells detected. **(I)** There was no significant change in the number of Sox2-positive, Hes5-LacZ-positive, or Sox2-positive/Hes5-LacZ-negative cells in the IPhCs/BCs region. ^∗∗^*p* < 0.01, ^∗∗∗^*p* < 0.001, ^∗∗∗∗^*p* < 0.0001 as determined by a two-way ANOVA with a Tukey’s or Sidak’s *post hoc* test. *N* = 4–5.

Because the majority of spontaneously regenerated HCs are formed in the apical turn of the cochlea (Cox et al., 2014), we investigated gene changes in this region at the cellular level. We first measured changes in the expression of *Hes5*, a direct inhibitor of HC fate that is a well-known target of active Notch signaling (Mulvaney and Dabdoub, 2012; Abdolazimi et al., 2016). *Hes5* is expressed in many SC subtypes during the first postnatal week including Deiters’ cells, outer pillar cells, inner phalangeal cells, border cells, and some cells of the greater epithelial ridge (GER) (**Figures 1A–C,G–I,M–O**, **2B–B”’,D–D”’,F–F”’**; Lanford et al., 2000; Zine et al., 2001; Hartman et al., 2009; Cox et al., 2014; Maass et al., 2015). To quantify the number of cells that express *Hes5*, we used the *Hes5^LacZ^* reporter line which has *LacZ* knocked into the endogenous *Hes5* locus and therefore is a faithful representation of endogenous *Hes5* expression (Imayoshi et al., 2010). Others have shown that β-galactosidase (β-gal), transcribed by the *LacZ* gene, continues to be expressed ∼1 day after mRNA of the endogenous gene becomes undetectable (Morrison et al., 1999). Therefore, we used *Hes5^LacZ^* mice to approximate the window when cells lose *Hes5* expression. *Atoh1-CreER^TM^::Rosa26^loxP-stop-loxP-DTA^* mice (hereinafter referred to as *Atoh1-DTA* mice) were bred with *Hes5^LacZ^* mice and injected with tamoxifen (3 mg/40 g, IP) on both P0 and P1 to induce HC ablation and initiate spontaneous HC regeneration. Samples were analyzed at P2, P4, and P6, which was previously established as the window of spontaneous HC regeneration seen in the neonatal mouse cochlea (Cox et al., 2014). We quantified the number of cells that expressed Hes5-LacZ as well as the number of SC and immature HC nuclei using anti-Sox2 antibodies. Significantly fewer Hes5-LacZ-positive cells were detected in the *Atoh1-DTA::Hes5^LacZ^* cochlea at P2 (216.1 ± 12.3 vs. 107.3 ± 19.1; *p* < 0.001; *N* = 3) and P4 (185.6 ± 13.3 vs. 115.5 ± 14.1; *p* < 0.01 as determined by a two-way ANOVA with a Tukey’s *post hoc* test; *N* = 3) compared to control samples that lacked either the *Atoh1-CreER^TM^* or *Rosa26^DTA^* allele (**Figures 1A–L,S**). However, there was no difference observed in Hes5-LacZ expression at P6 (140.1 ± 11.6 vs. 120.3 ± 7.3; *N* = 4; **Figures 1M–S**). There were also no differences in the number of Sox2-positive cells after HC damage at any age (**Figures 1B,E,H,K,N,Q,S**) suggesting that as a whole, the Sox2-positive cell population is not reduced.

Differences among SC subtypes have previously been documented (Lanford et al., 2000; Doetzlhofer et al., 2009; Yu et al., 2010; Taylor et al., 2012; Lagarde et al., 2013; Maass et al., 2016), which may indicate that not all SC subtypes maintain plasticity postnatally. Furthermore, loss of *Hes5* at embryonic ages has been implicated in the formation of supernumerary outer HCs, but not inner HCs (Zine et al., 2001). We therefore sought to investigate changes in *Hes5* expression within discrete SC subpopulations. To divide the SC subtypes into two groups, we used pillar and Deiters’ cells as a landmark. *S100a1* is a calcium binding protein that is expressed in the cytoplasm and nucleus of pillar and Deiters’ cells within the neonatal cochlea (White et al., 2006; Buckiová and Syka, 2009). Therefore, combining anti-S100a1 antibodies with anti-Sox2 and anti-β-gal antibodies allowed us to tease apart two groups of SC subpopulations: (1) pillar cells and Deiters’ cells which are labeled by S100a1, and (2) inner phalangeal and border cells which are *Hes5^LacZ^*-positive/Sox2-positive cells located medial to S100a1 labeling (**Figures 2A–B”’,D–D”’,F–F”’**). Because *Hes5^LacZ^* is also expressed in the cells of the GER, the medial border of inner phalangeal and border cells was established for quantification by measuring the width of tdTomato expression medial to inner pillar cells in *Plp-CreER^T2^::Rosa26^loxP-stop-loxP-tdTomato^* mice injected with tamoxifen (3 mg/40 g, IP) at P0, which has previously been shown to target inner phalangeal and border cells, but not the GER (**Supplementary Figures S1A–C**; Doerflinger et al., 2003; Gómez-Casati et al., 2010; Cox et al., 2012; Mellado Lagarde et al., 2014; McGovern et al., 2017).

After HC damage and regeneration was induced in *Atoh1-DTA::Hes5^LacZ^* mice by tamoxifen injection at P0 and P1, there was a significant decrease in the number of Hes5-LacZ-positive pillar and Deiters’ cells detected at P2 (113.1 ± 3.8 vs. 63.6 ± 5.1, *p* < 0.01, *N* = 4), P4 (104.4 ± 1.5 vs. 42.1 ± 5.7, *p* < 0.001, *N* = 4-5), and P6 (98.6 ± 2.4 vs. 25.3 ± 4.4, *p* < 0.001, *N* = 4) compared to control cochleae which lacked either the *Atoh1-CreER^TM^* or *Rosa26^DTA^* allele (**Figures 2B–H**). Of note, the number of Sox2-positive cells within this population did not decrease until P4 (140.2 ± 3.1 vs. 98.1 ± 11.5, *p* < 0.001; *N* = 4-5) and was also decreased at P6 (130.3 ± 2.7 vs. 75.0 ± 6.0, *p* < 0.0001; *N* = 4; **Figures 2B–H**). Additionally, there was an increase in the number of Sox2-positive/Hes5-LacZ-negative pillar and Deiters’ cells at P2 (39.0 ± 1.6 vs. 102.2 ± 21.9, *p* < 0.001; *N* = 4; **Figures 2B–H**), suggesting that *Hes5* is downregulated in these SCs, but the cells are still alive. In contrast, neither the number of *Hes5*-LacZ-positive cells, nor the number of Sox2-positive cells within the inner phalangeal and border cells region were significantly altered from control samples (**Figures 2B–G”’,I**), suggesting that weakened Notch signaling is limited to pillar and Deiters’ cells during the HC regeneration process.

### Reduced Expression of Notch Effectors *Hes1, Hes5, Hey1*, and *HeyL* Was Observed During Spontaneous HC Regeneration

**FIGURE 3:**
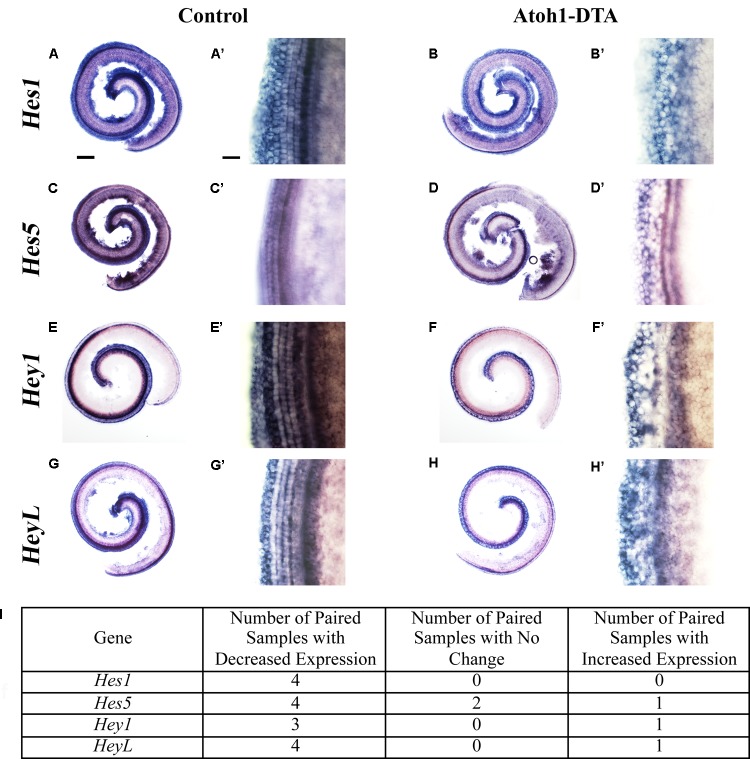
*Hes1*, *Hes5*, *Hey1*, and *HeyL* are decreased during spontaneous HC regeneration. **(A–B’)** *Hes1*, **(C–D’)** *Hes5*, **(E–F’)** *Hey1*, and **(G–H’)** *HeyL* transcripts were investigated during spontaneous HC regeneration using *in situ* hybridization. Low magnification images show the whole cochlea and high magnification images were taken from the apical turn where the majority of spontaneous HC regeneration occurs. Control cochlea **(A,A’,C,C’,E,E’,G,G’)** as well as *Atoh1-DTA* **(B,B’,D,D’,F,F’,H,H’)** littermates were injected with tamoxifen (Tam) at P0 and P1 to induce HC damage and spontaneous HC regeneration. Samples were collected at P4 and incubated with probes to label mRNA of Notch downstream effectors. Paired samples from the same litter (control and *Atoh1-DTA*) were processed in parallel and comparisons were only made between the paired samples. While there was some variability in the results obtained among experiments **(I)**, the majority of experiments showed decreased gene expression for all four Notch effectors in *Atoh1-DTA* cochleae compared to control. Scale bar for **(A,B,C,D,E,F,G,H)** = 200 μm, Scale bar for **(A’,B’,C’,D’,E’,F’,G’,H’)** = 20 μm. *N* = 4–7.

Further changes in the expression of Notch target genes were investigated using *in situ* hybridization. *Atoh1-DTA* mice as well as littermate controls, which lacked the *Atoh1-CreER^TM^* or *Rosa26^DTA^* allele, were injected with tamoxifen (3 mg/40 g, IP) on P0 and P1 to induce HC damage as well as spontaneous HC regeneration. Samples were collected and analyzed at P4 using probes that target *Hes1*, *Hes5*, *Hey1*, and *HeyL*. Paired samples from the same litter (control and *Atoh1-DTA*) were processed in parallel where each solution, as well as each incubation time, was identical. Comparisons were only made between the one experimental and the one control sample that were processed together. Staining intensity in the sensory region (low magnification images in **Figures 3A,B,C,D,E,F,G,H**) and at the HC level (high magnification images taken in the apical turn, **Figures 3A’,B’,C’,D’,E’,F’,G’,H’**) was compared between the paired control and experimental samples to determine increased or decreased expression. Expression of *Hes1* was consistently decreased in *Atoh1-DTA* samples compared to controls (**Figure 3A–B’,I**, *N* = 4), while variable results were obtained for the other tested genes. For *Hes5*, four out of seven samples showed decreased expression, while one showed increased expression and two showed no change in expression (**Figures 3C–D’,I**). *Hey1* expression was also variable with three samples showing decreased expression and one sample showing increased expression (**Figures 3E–F’,I**). For *HeyL*, four samples showed decreased expression, while one showed increased expression (**Figures 3G–H’,I**). SC-to-HC conversion is a dynamic and non-synchronous process that occurs throughout the first postnatal week (Cox et al., 2014); the variability of individual gene changes we observed may depend on the stage of SC conversion into HCs at the time of analysis. Nevertheless, because the majority of the samples showed decreased expression of Notch effectors, we conclude that loss of HCs in the neonatal mouse cochlea caused a modest decrease in Notch signaling in neighboring SCs.

### The Notch Ligand and Target Gene *Jag1* Is Reduced During Spontaneous HC Regeneration

**FIGURE 4:**
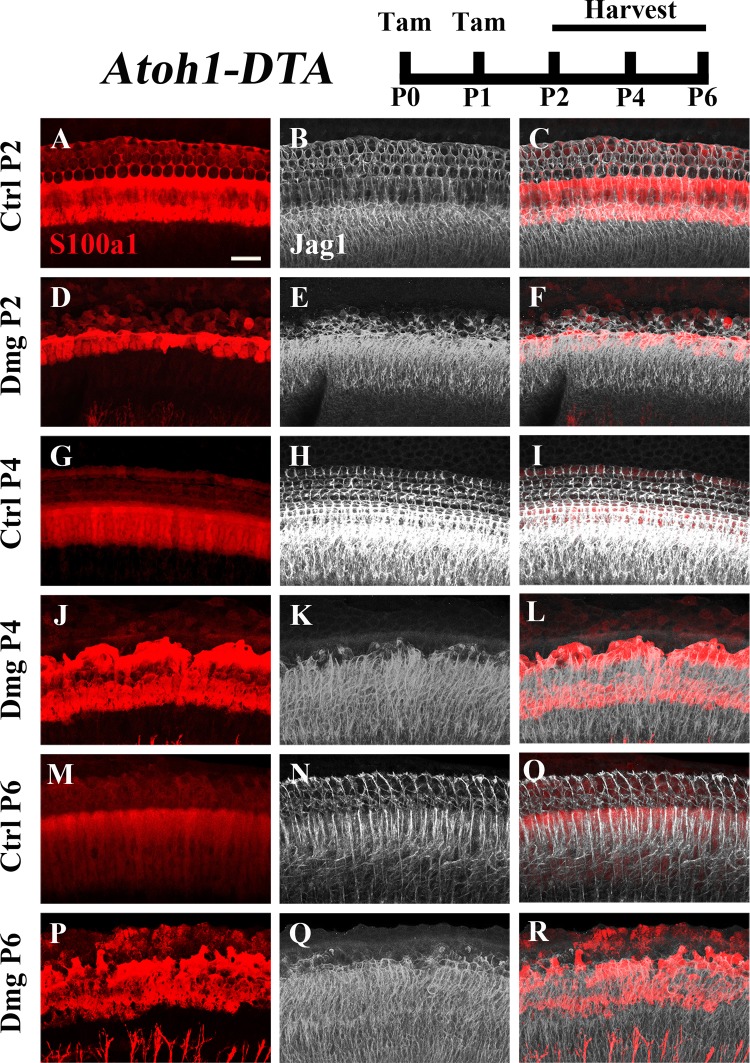
Jag1 labeling is reduced in lateral, but not medial, SCs during spontaneous HC regeneration. *Atoh1-DTA* mice were injected with tamoxifen (Tam) at P0–P1 to induce HC death and spontaneous HC regeneration and cochleae were collected at P2, P4, or P6. **(A,R)** Representative maximum projection images with anti-Jag1 antibodies to detect Jag1 expression (white) and anti-S100a1 (red) antibodies to mark the pillar and Deiters’ cells region. After HC damage, both S100a1 and Jag1 labeling was reduced in the outer pillar and Deiters’ cell region, while expression is maintained in medial SCs **(D–F,J–L,P–R)** and in all SCs in control samples **(A–C,G–I,M–O)**. Scale bar = 25 μm. *N* = 3.

The Notch ligand *Jag1* is expressed by neonatal SCs and is also a downstream target of Notch activation (Daudet and Lewis, 2005; Murata et al., 2009). Therefore, it can be used as an indicator of active Notch signaling. Early in development *Jag1* mediates lateral induction, establishing the progenitor pool from which all HCs and SCs differentiate (Brooker et al., 2006; Kiernan et al., 2006; Murata et al., 2009). However, its function during and after HC differentiation is not well understood, yet it is expressed throughout the organ of Corti from the cells of the GER to the 3rd row of Deiters’ cells (**Figures 4A–C,G–I,M–O**; Oesterle et al., 2008). We investigated changes in *Jag1* in the context of HC loss and spontaneous HC regeneration as an additional way to assess changes in the Notch signaling pathway. Specifically, *Atoh1-DTA* mice were injected with tamoxifen (3 mg/40 g, IP) at P0 and P1 to induce HC ablation thus initiating spontaneous HC regeneration. Samples were collected at P2, P4, and P6 and immunolabeled with antibodies against S100a1 and Jag1. Because Jag1 is a membrane bound protein, we could not determine which neighboring cells expressed Jag1 and therefore quantitative analysis was not performed. However, qualitative analysis of *Atoh1-DTA* cochleae showed that Jag1 labeling was reduced in the lateral region of the organ of Corti, where outer pillar cells and Deiters’ cells are located, yet maintained in medial SCs, including inner pillar cells, inner phalangeal/border cells, and the cells of the GER (**Figures 4D–F,J–L,P–R**). In contrast, Jag1 labeling was consistent in all SCs throughout the width of organ of Corti in control mice (**Figures 4A–C,G–I,M–O**). These data confirm the results obtained using the *Hes5^LacZ^* reporter mice and demonstrate that Notch signaling is reduced in some SC subtypes, but not others, during spontaneous HC regeneration.

### Overexpression of *N1ICD* Caused Increased Notch Activity in the Neonatal Cochlea

While immunostaining and *in situ* hybridization results suggest a modest decrease in Notch signaling during spontaneous HC regeneration, it is currently unknown whether Notch signaling is involved in the mechanism of spontaneous HC regeneration or if it is simply correlated. To investigate this question more directly, we induced overexpression of *N1ICD* in SCs and other non-sensory epithelial cells (all other cells within the organ of Corti except for HCs and tympanic border cells, hereinafter referred to as non-HCs) in the context of HC damage and quantified the number of regenerated HCs using fate-mapping. This was achieved by combining CreER/loxP and Tet-on genetically modified mouse systems to modify gene expression in adjacent cell populations.

**FIGURE 5:**
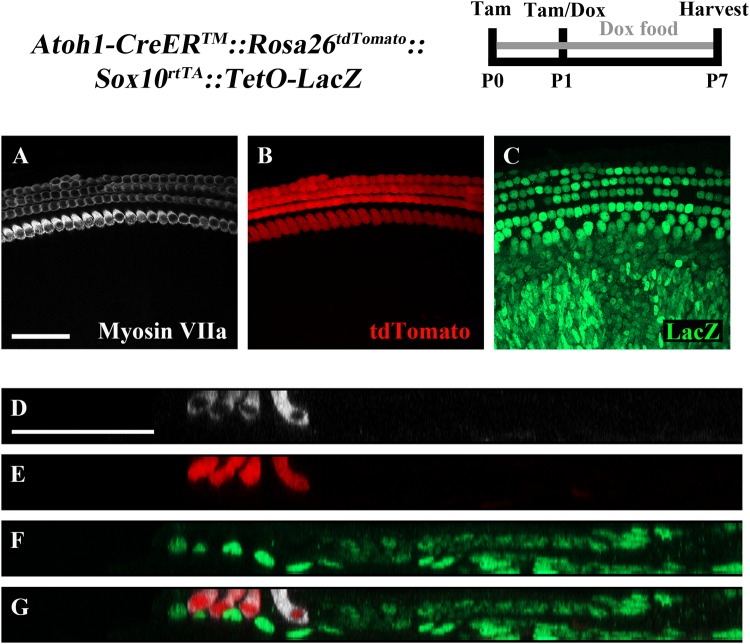
The combination of CreER/loxP and Tet-On genetic systems allow gene manipulation in distinct cell types without cross-reactivity. *Atoh1-CreER^TM^*::*Rosa26^loxP-stop-loxP-tdTomato^::Sox10^rtTA^::TetO-LacZ* mice were injected with tamoxifen (Tam) at P0 and P1 to induce tdTomato expression. Doxycycline (Dox) was administered to nursing mother in the diet and each pup received a dox injection at P1 to induce LacZ expression. Cochleae were collected at P7 and stained with antibodies against myosin VIIa (white) to label HCs and β-gal (green) to label LacZ-positive cells. **(A–C)** Representative confocal slice images show expression of endogenous tdTomato fluorescence (red) detected in HCs similar to Cox et al. (2014). β-gal expression (green) was detected in SCs and other non-HCs similar to Walters and Zuo (2015). **(D–G)** Optical cross-sections showing expression of tdTomato in HCs and β-gal in SCs and all non-HCs. Scale bar = 50 μm. *N* = 3.

To ensure feasibility of using these two inducible systems in adjacent groups of cells without cross-reaction, dual reporter expression was investigated in *Atoh1-CreER^TM^::Rosa26^loxP-stop-loxP-tdTomato^ ::Sox10^rtTA^ ::TetO-LacZ* mice. tdTomato expression was induced with tamoxifen injection (3 mg/40 g, IP) at P0 and P1, while LacZ expression was induced by administering doxycycline to the nursing mother in the food (2000 mg/kg) from P0–P7, as well as a single injection (100 mg/kg) to each pup at P1. Samples were analyzed at P7. As expected, tdTomato expression was only detected in HCs, while LacZ expression was observed in SCs and non-HCs (**Figures 5A–G**). We also observed variability in the intensity of LacZ expression cell to cell, which was similar to the previously described pattern using the same doxycycline induction paradigm in *Sox10^rtTA^::TetO-LacZ* mice (Walters and Zuo, 2015).

**FIGURE 6:**
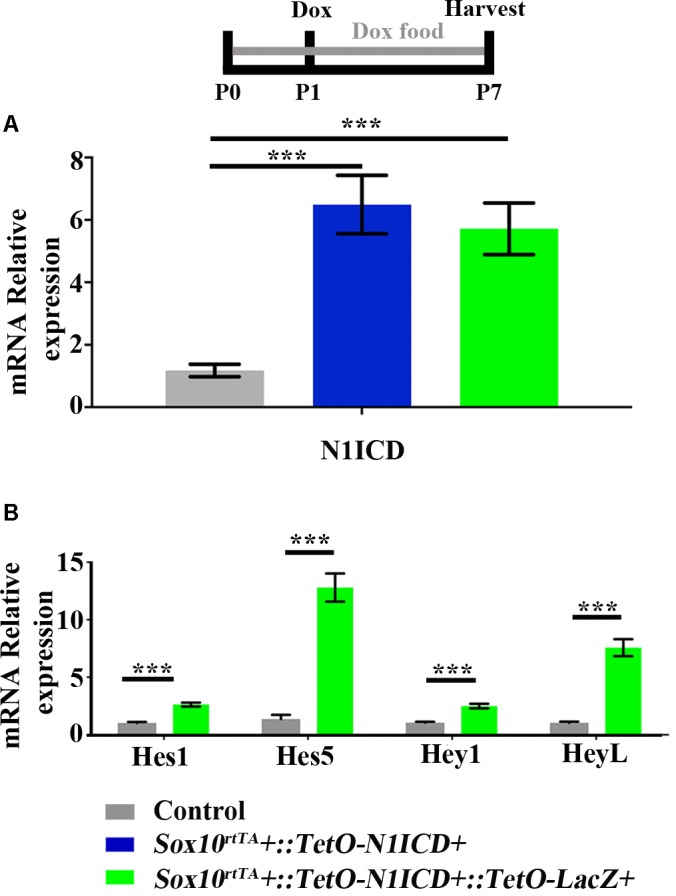
Increased expression of *N1ICD* driven by *Sox10^rtTA^* leads to increased expression of Notch downstream effectors. *Sox10^rtTA^-*negative, *Sox10^rtTA^::TetO-N1ICD*, and *Sox10^rtTA^::TetO-LacZ::TetO-N1ICD* mice were administered doxycycline (Dox), in the diet to nursing mother, as well as an injection to each pup at P1. Cochleae were frozen at P7 and mRNA transcripts were measured. **(A)** *N1ICD* expression was increased in *Sox10^rtTA^::TetO-N1ICD*, and *Sox10^rtTA^::TetO-LacZ::TetO-N1ICD* cochlea, compared to *Sox10^rtTA^-*negative controls. There was no difference in the expression of *N1ICD* when *TetO-LacZ* was also present. **(B)** Expression level of Notch effectors *Hes1, Hes5, Hey1*, and *HeyL* were increased in *Sox10^rtTA^::TetO-LacZ::TetO-N1ICD* cochlea, compared to *Sox10^rtTA^-*negative controls. ^∗∗∗^*p* < 0.001 as determined by a one-way ANOVA with a Dunnett’s *post hoc* test or a Student’s *t*-test. *N* = 10.

To investigate whether downregulation of Notch signaling plays a role in spontaneous HC regeneration, we used *Sox10^rtTA^*::*TetO-LacZ*::*TetO-N1ICD* mice to overexpress *N1ICD* as well as *LacZ* to fate-map cells. Therefore, in this mouse model, *Sox10^rtTA^* must activate two Tet-on alleles simultaneously. To verify that active Notch signaling was achieved in our model and that the presence of the *TetO-LacZ* allele did not affect the level of Notch activation, we measured *N1ICD* expression levels using qPCR. Specifically, *Sox10^rtTA^*-negative, *Sox10^rtTA^*::*TetO-N1ICD*, and *Sox10^rtTA^*::*TetO-LacZ*::*TetO-N1ICD* mice were administered doxycycline, both in the diet to the nursing mother (2000 mg/kg), as well as a single injection to the pups (100 mg/kg, IP) at P1. RNA was extracted from samples collected at P7 and *N1ICD* expression was analyzed with qPCR. Of note, the primers used for *N1ICD* detection recognize both the endogenous and transgenic transcripts and data is expressed as fold change relative to the *Sox10^rtTA^*-negative control. In both *Sox10^rtTA^*::*TetO-N1ICD* and *Sox10^rtTA^*::*TetO-LacZ*::*TetO-N1ICD* groups, *N1ICD* was increased 5 to 6-fold (6.4 ± 0.9-fold for *Sox10^rtTA^*::*TetO-N1ICD, p* < 0.001, *N* = 10 and 5.7 ± 0.8-fold for *Sox10^rtTA^*::*TetO-LacZ*::*TetO-N1ICD, p* < 0.001, *N* = 10 compared to *Sox10^rtTA^*-negative samples; as determined by a one-way ANOVA with a Tukey’s *post hoc* test, **Figure 6A**). There was no difference in the expression level of *N1ICD* between *Sox10^rtTA^*::*TetO-N1ICD* and *Sox10^rtTA^*::*TetO-LacZ*::*TetO-N1ICD* samples, indicating that the addition of the *TetO-LacZ* allele did not interfere with increased Notch activity induced by the *TetO-N1ICD* allele.

To further confirm increased Notch activity in our model, we used qPCR to investigate the expression level of Notch target genes, *Hes1*, *Hes5*, *Hey1*, and *HeyL*. All Notch effector genes were increased 2 to 13-fold in *Sox10^rtTA^*::*TetO-LacZ*::*TetO-N1ICD* cochleae compared to *Sox10^rtTA^-*negative controls (*Hes1* increased 2.6 ± 0.1-fold, *p* < 0.0001, *N* = 10; *Hes5* increased 12.7 ± 1.2-fold, *p* < 0.0001, *N* = 10; *Hey1* increased 2.4 ± 0.2-fold, *p* < 0.0001, *N* = 10; and *HeyL* increased 7.5 ± 0.7-fold, *p* < 0.0001, *N* = 10 compared to *Sox10^rtTA^*-negative controls as determined by a Student’s *t*-test for each gene; **Figure 6B**). Therefore, we conclude that Notch signaling was increased by *N1ICD* overexpression in our model.

### Spontaneous HC Regeneration Is Reduced When the *N1ICD* Is Overexpressed in SCs and Other Non-HCs

To investigate whether spontaneous HC regeneration could be prevented by increased Notch signaling, we overexpressed *N1ICD* in SCs and other non-HCs in the context of HC damage using *Atoh1-DTA::Sox10^rtTA^::TetO-LacZ::TetO-N1ICD* mice. The effect of increased Notch signaling on spontaneous HC regeneration was investigated by quantifying the number of fate-mapped SCs. Specifically, we analyzed the number of LacZ-positive HCs in each of four groups. First, to verify the expression pattern of *Sox10^rtTA^* in a normal neonatal cochlea, the number of LacZ-positive HCs was quantified in *Sox10^rtTA^*::*TetO-LacZ* mice. Second, to determine the number of spontaneously regenerated HCs that occur naturally in the neonatal mouse cochlea after HC loss at birth, we quantified LacZ-positive HCs in *Atoh1-DTA::Sox10^rtTA^*::*TetO-LacZ* mice. Third, to determine the effect of *N1ICD* overexpression in SCs and other non-HCs in the neonatal mouse cochlea, LacZ-positive HCs were quantified in *Sox10^rtTA^*::*TetO-LacZ*::*TetO-N1ICD* mice. Finally, to investigate whether overexpression of *N1ICD* in SCs and other non-HCs will prevent spontaneous HC regeneration from occurring, we quantified LacZ-positive HCs in *Atoh1-DTA::Sox10^rtTA^*::*TetO-LacZ*::*TetO-N1ICD* mice. Mice from each group were injected with tamoxifen (3 mg/kg, IP) at P0 and P1 to induce HC damage. *N1ICD* and *LacZ* expression were induced through administration of doxycycline given both in the food (2000 mg/kg) to the nursing mother, as well as one doxycycline injection (100 mg/kg, IP) given to the pups at P1, ∼6 h after tamoxifen injection. Samples were collected at P7 and processed for immunostaining with antibodies against myosin VIIa, Sox2, and β-gal.

**FIGURE 7:**
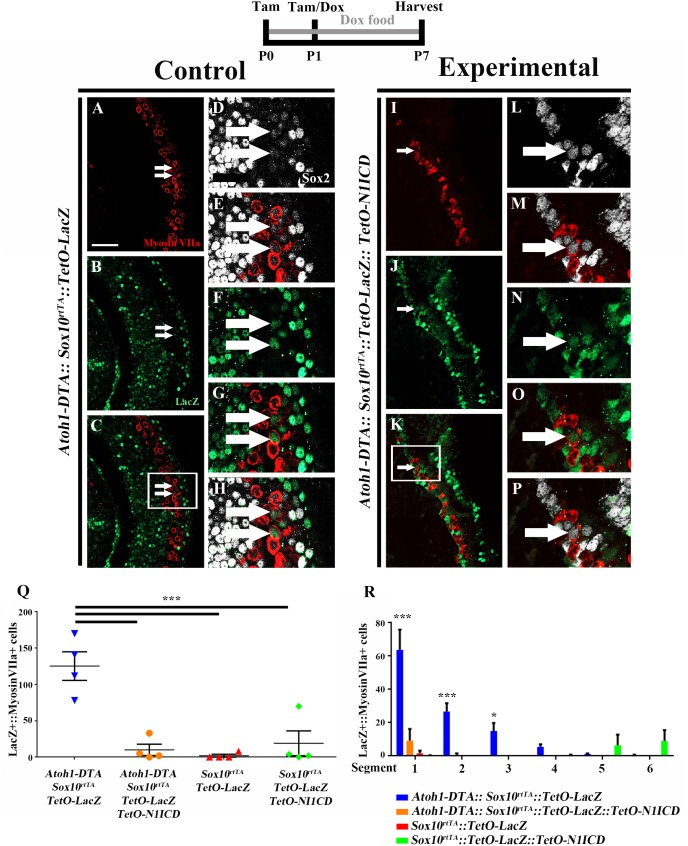
Fewer spontaneously regenerated HCs were observed in the presence of *N1ICD* overexpression. Representative confocal slice images taken from the apical turn of the cochlea of **(A–H)** *Atoh1-DTA::Sox10^rtTA^::TetO-LacZ* and **(I–P)** *Atoh1-DTA::Sox10^rtTA^::TetO-LacZ::TetO-N1ICD* mice that were injected with tamoxifen (Tam) at P0/P1 to induce CreER-mediated HC loss and induce spontaneous HC regeneration, as well as administered doxycycline (Dox) in the diet to nursing mother with a dox injection given to pups at P1 to induce expression of *N1ICD* and the *LacZ* reporter in SCs and all non-HCs. Cochlea were collected at P7 and stained with antibodies against myosin VIIa (red), Sox2 (white), and β-gal (green). Fewer fate-mapped regenerated HCs (LacZ-positive/myosin VIIa-positive cells, white arrows) were observed when *N1ICD* was overexpressed in combination with HC damage compared to samples with HC damage and normal Notch signaling. **(D–H**,**L–P)** High magnification images from boxes in **(C,K)** showing LacZ-positive/Sox2-positive/myosin VIIa-positive regenerated HCs. Scale bar = 25 μm. **(Q)** Quantification of LacZ-positive HCs from the images presented in **(A–P)** and the two additional controls. Comparison of the number of LacZ-positive HCs between the group with spontaneous HC regeneration and normal Notch signaling (blue, *Atoh1-DTA::Sox10^rtTA^::TetO-LacZ* mice) and the experimental group with *N1ICD* overexpression as well as HC damage (orange, *Atoh1-DTA::Sox10^rtTA^::TetO-LacZ::TetO-N1ICD)*, showed a ∼92% decrease in the number of LacZ-positive, regenerated HCs. **(R)** LacZ-positive HCs after HC damage with physiological Notch signaling (blue, *Atoh1-DTA::Sox10^rtTA^::TetO-LacZ*) decreased in an apical to basal gradient while LacZ-positive HCs were only detected in the base when *N1ICD* was overexpressed without HC damage (green, *Sox10^rtTA^::TetO-LacZ::TetO-N1ICD*) mice. Segment 1 is the most apical region and segment 6 is the most basal. *N* = 3–4. ^∗^*p* < 0.05, ^∗∗∗^*p* < 0.001 as determined by a two-way ANOVA with a Tukey’s *post hoc* test. *N* = 4.

As expected from previous work (Walters and Zuo, 2015), *LacZ* expression in *Sox10^rtTA^*::*TetO-LacZ* mice was observed in SCs and other non-HCs within the P7 cochlea. We also observed 2.0 ± 2.0 LacZ-positive HCs in the very apical tip (**Figures 7Q,R**, *N* = 4). There was no significant change in LacZ-positive HCs when *N1ICD* was overexpressed without HC damage in *Sox10^rtTA^*::*TetO-LacZ*::*TetO-N1ICD* mice (19.0 ± 17.2, compared to *Sox10^rtTA^*::*TetO-LacZ* controls, *N* = 4, *p* = 0.8 as determined by a one-way ANOVA with a Tukey’s *post hoc* test; **Figure 7Q**). However, we observed disorganization of HCs (**Supplementary Figures S2A,G**) as well as an expansion of Sox2 expression both medial and lateral to the organ of Corti, (**Supplementary Figures S2A–D,F–I**). Quantification of HCs in *Sox10^rtTA^*::*TetO-LacZ*::*TetO-N1ICD* mice showed that there was no difference in the number of HCs compared to *Sox10^rtTA^*::*TetO-LacZ* mice (91.0 ± 2.5 compared to 88.7 ± 5.9 per 200 μm, *p* = 0.5 as determined by a Student’s *t*-test, **Supplementary Figure S2E**) and therefore this phenotype likely did not affect spontaneous HC regeneration observed in our study.

When LacZ was used for fate-mapping during spontaneous HC regeneration, significantly more LacZ-positive HCs were detected in *Atoh1-DTA::Sox10^rtTA^*::*TetO-LacZ* mice than in *Sox10^rtTA^*::*TetO-LacZ* controls (125.3 ± 19.7 compared to 2.0 ± 2.0, *p* = 0.0002, *N* = 4, **Figure 7Q**). When *N1ICD* was overexpressed in the context of HC damage (*Atoh1-DTA::Sox10^rtTA^*::*TetO-LacZ*::*TetO-N1ICD* mice), there was a 92% reduction in LacZ-positive HCs compared to similar samples without *N1ICD* overexpression (*Atoh1-DTA::Sox10^rtTA^*::*TetO-LacZ* mice) (10.0 ± 7.7 compared to 125.3 ± 19.7, *p* = 0.0003, *N* = 4, **Figures 7A–Q**). Moreover, the number of LacZ-positive HCs when *N1ICD* was overexpressed and HCs were killed was not significantly different from controls lacking both HC damage and *N1ICD* overexpression (*Sox10^rtTA^*::*TetO-LacZ* samples) (10.0 ± 7.7 compared to 2.0 ± 2.0, *p* = 0.9) or *N1ICD* overexpression alone (*Sox10rtTA*::*TetO-LacZ*::*TetO-N1ICD* samples) (10.0 ± 7.7 compared to 19.0 ± 17.0, *p* = 0.9 as determined by a one-way ANOVA with a Tukey’s *post hoc* test; **Figure 7Q**). Therefore, we conclude that overexpression of *N1ICD* prevented the majority of spontaneous HC regeneration from occurring. Of note, we observed an expansion of Sox2 expressing cells in *Atoh1-DTA::Sox10^rtTA^*::*TetO-LacZ*::*TetO-N1ICD* mice, similar to what we observed in *Sox10^rtTA^*::*TetO-LacZ*::*TetO-N1ICD* controls (**Supplementary Figures S2F–M**).

We wanted to better understand the location of fate-mapped, spontaneously regenerated HCs in our samples because the majority of spontaneous HC regeneration is known to occur in the apical third of the neonatal cochlea (Cox et al., 2014) and because Notch signaling decreases in a basal to apical gradient during the first postnatal week (Maass et al., 2015). Therefore, the whole cochlea was imaged, measured, and divided into six equal segments from apex to base as previously described (McGovern et al., 2017) and the total number of LacZ-positive/myosin VIIa-positive HCs within each segment was quantified. The majority of fate-mapped, spontaneously regenerated HCs (from *Atoh1-DTA::Sox10^rtTA^*::*TetO-LacZ* mice) were found in the two most apical segments (63.8 ± 12.0 in segment 1 and 26.8 ± 4.9 in segment 2) and this declined in an apical to basal gradient (15.4 ± 4.8 in segment 3, 5.6 ± 1.4 in segment 4, 1.2 ± 0.3 in segment 5, and 0.4 ± 0.4 in segment 6, *r*^2^ = 0.66. *p* < 0.0001 **Figure 7R**), similar to the previous report (Cox et al., 2014). In one S*ox10^rtTA^*::*TetO-LacZ*::*TetO-N1ICD* sample that did not have HC damage, we observed 67 LacZ-positive HCs in the two basal segments, but no LacZ-positive HCs were detected in the middle or apical segments of the cochlea where the majority of HC regeneration was observed (**Figure 7R**). The other three S*ox10^rtTA^*::*TetO-LacZ*::*TetO-N1ICD* samples had no LacZ-positive HCs. Therefore, the LacZ-positive HCs observed in *N1ICD* overexpressing controls did not interfere with the quantification of fate-mapped, spontaneously regenerated HCs predominantly observed in the apical turn of the cochlea. When *N1ICD* was overexpressed along with HC damage, the fate-mapped regenerated HCs in the *Atoh1-DTA::Sox10^rtTA^*::*TetO-LacZ*::*TetO-N1ICD* mice were all located within the first two apical segments (**Figure 7R**), similar to the control with spontaneous HC regeneration and no manipulation of Notch (*Atoh1-DTA::Sox10^rtTA^*::*TetO-LacZ* mice). This illustrates that the regenerative capacity of SCs is correlated to the basal to apical changes in the Notch signaling pathway. Our data show weakened Notch signaling during the spontaneous HC regeneration process, as well as the suppression of HC regeneration by increased Notch activity. Taken together, this suggests that Notch signaling plays a role in the ability of SCs to spontaneously convert into, thus regenerate, HCs.

## Discussion

This study investigated the role of Notch signaling during spontaneous HC regeneration within the neonatal mouse cochlea *in vivo*. After HC damage and during the window of spontaneous HC regeneration, we observed a modest decrease in Notch signaling, as evidenced by decreased expression of the Notch target genes *Hes1, Hes5, Hey1, HeyL*, and *Jag1* using immunostaining or *in situ* hybridization. These changes suggest that Notch signaling is weakened in SCs after HC loss occurs and may allow their conversion into HCs. To investigate this further, we increased Notch signaling in SCs and other non-HCs by overexpressing *N1ICD* during the window of spontaneous HC regeneration. In samples with increased Notch signaling, there was a 92% reduction in the number of spontaneously regenerated HCs, suggesting that Notch signaling plays a role in spontaneous HC regeneration.

### The Expression of *Hes1*, *Hes5*, *Hey1*, and *HeyL* Were Reduced During the Window of Spontaneous HC Regeneration

Using *in situ* hybridization and a *Hes5^LacZ^* knockin reporter line, we discovered that the expression of *Hes1, Hes5, Hey1*, and *HeyL* was reduced after HC loss and during the spontaneous HC regeneration process. Both *Hes1* and *Hes5* directly inhibit a HC fate by inhibiting the expression of *Atoh1*, a transcription factor that is necessary for HC differentiation (Zheng et al., 2000; Mulvaney and Dabdoub, 2012; Abdolazimi et al., 2016). Previously, Korrapati et al. (2013) found that HC damage after gentamicin treatment *in vitro* leads to downregulation of *Hes5*, but no other Notch-related genes expressed in SCs. However, no HC regeneration was observed in this model until Notch signaling was inhibited with a γ-secretase inhibitor, which caused decreased expression of all Notch downstream effectors. This suggests that reduction of *Hes5* signaling alone is insufficient to induce spontaneous HC regeneration, however, when reduced expression is expanded to additional Notch effectors, regeneration is possible. In support, overexpression of *Hes1* is able to inhibit the production of ectopic HCs in the GER that were created by ectopic expression of *Atoh1.* This indicates that *Hes1* is also a direct inhibitor of HC fate (Zheng et al., 2000). Furthermore, combined loss of *Hes1*, *Hes5*, and *Hey1* during late embryonic development [embryonic day (E) 18.5] caused increased outer HCs with disorganized pillar and Deiters’ cells (Tateya et al., 2011). Unfortunately, less is known about the function of the Notch effector *HeyL* in cochlear development, however, it is expressed around the time of HC differentiation and is linked to neuronal differentiation in other systems (Doetzlhofer et al., 2009; Jalali et al., 2011). Therefore, combined reduction of *Hes1, Hes5*, and *Hey1*, and possibly *HeyL*, observed in our study are correlated with the ability of SCs to spontaneously regenerate HCs in the neonatal mouse cochlea, thus supporting our conclusion that weakened Notch signaling allows SC-to-HC conversion and plays a role in spontaneous HC regeneration.

### Jag1 Is Reduced During the Spontaneous HC Regeneration Process

In the developing cochlea, *Jag1* is initially expressed throughout the sensory epithelium, then restricted to SCs, with expression in all SC subtypes from the GER to the 3rd row of Deiters’ cells at neonatal ages and in adulthood (Morrison et al., 1999; Daudet and Lewis, 2005; Oesterle et al., 2008; Murata et al., 2009). However, its function during and after HC differentiation is not well understood. Furthermore, *Jag1* is both a Notch ligand as well as a Notch target gene (Daudet and Lewis, 2005; Murata et al., 2006) and as such, reduced expression of this gene can indicate loss of Notch signaling. Consistent with results from the *Hes5^LacZ^* reporter and *in situ* hybridization, Jag1 expression was decreased in *Atoh1-DTA* samples in outer pillar and Deiters’ cells, the SC subtypes located underneath outer HCs in the lateral compartment of the cochlea. This finding further supports our conclusion that there is a modest decrease in Notch activity in SCs after HC damage and during HC regeneration in the neonatal cochlea.

### The Majority of Spontaneous HC Regeneration Is Prevented When *N1ICD* Is Overexpressed in SCs and Other Non-HCs in the Neonatal Mouse Cochlea

Previous work has focused on the effect of induced Notch inhibition via pharmacologic manipulation with γ-secretase inhibitors (Yamamoto et al., 2006; Korrapati et al., 2013; Li et al., 2015) or deletion of Notch receptors or *RBP-J* (Yamamoto et al., 2006; Bramhall et al., 2014; Li et al., 2015). From these studies we know that loss of Notch signaling triggers SC-to-HC conversion, however, while this approach produces new HCs, it applies a blanket reduction in all Notch related genes, as well as potentially affects other signaling pathways that γ-secretase interacts with such as ErbB-4, E-Cadherin, N-Cadherin, EphrinB, and CD44 (Ni et al., 2001; Lammich et al., 2002; Marambaud et al., 2002, 2003; Georgakopoulos et al., 2006). Therefore, it is unclear whether decreased Notch signaling in SCs is part of the molecular mechanism for spontaneous HC regeneration. Our study attempts to address this gap in knowledge by showing that overexpression of *N1ICD* in SCs and other non-HCs causes an increase in Notch signaling and results in a 92% reduction in the number of regenerated HCs. However, the mouse model we used generated supraphysiological levels of Notch activity with *N1ICD* increased 5 to 6-fold and *Hes*/*Hey* genes increased 2 to 13-fold over controls. It is possible that the level of Notch signaling is important in controlling HC and SC fate. In support of this notion, there was one *Atoh1-DTA::Sox10^rtTA^*::*TetO-LacZ*::*TetO-N1ICD* sample with 33 fate-mapped regenerated HCs, while the other three samples that had 0-5 fate-mapped regenerated HCs. While not directly tested, the sample with more regenerated HCs may have had lower expression of *N1ICD* compared to the other three samples, which could have been caused by decreased exposure to doxycycline. Despite the limitation of supraphysiological *N1ICD* and Notch effector levels in our model, data from this study support a role for Notch signaling during the spontaneous HC regeneration process.

*Sox10^rtTA^* is a knockin allele that may produce decreased expression of *Sox10* in the neonatal cochlea. It is possible that decreased *Sox10* expression may alter normal spontaneous HC regeneration making it more dependent on Notch signaling and therefore making *N1ICD* overexpression more effective at preventing regeneration. While we cannot rule out this possibility, a previous study showed that *Sox10^rtTA^* mice do not have a haploinsufficient phenotype in the cochlea (Walters and Zuo, 2015). Yet little is known about the relationship between *Sox10* and Notch signaling, and further research is needed.

Unexpectedly, in the control samples where *N1ICD* was overexpressed without HC damage (*Sox10^rtTA^::TetO-LacZ::TetO-N1ICD* mice) and in experimental samples (*Atoh1-DTA::Sox10^rtTA^*::*TetO-LacZ*::*TetO-N1ICD* mice), we observed an expansion of the region with Sox2-positive cells both medial and lateral to the organ of Corti. *Sox10^rtTA^::TetO-LacZ::TetO-N1ICD* controls also had disorganized HCs, but no decrease in HC number. Similar studies were done previously in the embryonic and early neonatal cochlea where *N1ICD* overexpression led to ectopic sensory patches containing both Sox2-positive cells and HCs in non-sensory regions. Yet this was only observed when *N1ICD* was overexpressed prior to E14.5 (Hartman et al., 2010; Pan et al., 2010, 2013; Liu et al., 2012; Campbell et al., 2015). Studies where *N1ICD* overexpression was induced after E14.5 showed no ectopic HCs or Sox2-positive cells (Liu et al., 2012; Pan et al., 2013). These studies demonstrate that *N1ICD* overexpression can induce Sox2 expression and sensory progenitor cells in non-sensory regions, yet there is a temporal limitation for when these cells can respond. Therefore, the increased numbers of Sox2-positive cells we observed when *N1ICD* was overexpressed at P0 was unexpected. We may have overcome temporal limitations by producing supraphysiological levels of *N1ICD* and Notch effectors, but further experiments are needed to confirm this hypothesis. Regardless of the cause, we do not believe expanded Sox2 expression altered the spontaneous HC regeneration process or affected the conclusions of our study since all abnormalities, except HC disorganization, were observed outside the organ of Corti.

Notch signaling changes dynamically in the first week after birth where ligands, receptors, and effectors are downregulated in a basal-to-apical gradient between P0 and P6 (Murata et al., 2006; Hartman et al., 2007; Maass et al., 2015). However, Jag1 is the exception and its expression in SCs continues throughout adulthood (Oesterle et al., 2008). During the first postnatal week, inhibition of Notch signaling using γ-secretase inhibitors has differential effects on SC-to-HC conversion along the basal to apical axis. Notch inhibition at P0 produces a ∼200% increase in HCs in the apex compared to only a ∼30% increase in the base, while there is no evidence of HC production in any cochlear turn when γ-secretase inhibitors are used at P3 or later (Maass et al., 2015). There are conflicting reports for whether SCs can produce HCs in response to Notch inhibition in the adult cochlea (Batts et al., 2009; Doetzlhofer et al., 2009; Hartman et al., 2009; Mizutari et al., 2013). These data taken together with our results suggest that spontaneous HC regeneration likely occurs when HCs are killed at P0–P1 because SCs, especially those in the apical turn, are still dependent on Notch signaling to maintain their cell fate. As the cochlea matures, it appears that SC fate maintenance becomes uncoupled from Notch signaling, yet the upstream regulators or epigenetic changes that cause downregulation of the Notch pathway, as well as the mechanisms that maintain SC fate after the first postnatal week are unknown.

Nevertheless, while previous studies have shown that increased numbers of regenerated HCs can form when Notch signaling is inhibited (Korrapati et al., 2013; Bramhall et al., 2014; Hu et al., 2016), the findings presented here are the first to demonstrate that the spontaneous HC regeneration process is prevented by increased Notch signaling. This suggests that weakened Notch signaling plays a role in the spontaneous HC regeneration process. Understanding the molecular mechanism of spontaneous HC regeneration is a critical step to understanding why HC regeneration does not occur in the mature cochlea. Additionally, while some Notch effectors, such as *Hes1* and *Hes5*, are well established as inhibitors of HC fate, less is known about the contribution of other effectors on SC-to-HC conversion. The findings presented here will hopefully drive research to identify cellular and molecular changes that occur within the first postnatal week to uncouple Notch signaling and SC fate maintenance. Understanding what assumes control over SC fate in the adult cochlea will potentially lead investigations into therapeutic targets to stimulate HC regeneration in the mature cochlea.

## Author Contributions

MM designed and performed the experiments, analyzed the data, and wrote the manuscript. LZ and MR performed the experiments, analyzed the data, and contributed to writing the manuscript. BC designed the experiments, analyzed the data, and wrote the manuscript.

## Conflict of Interest Statement

BC is a consultant and MR is a part-time employee of Turner Scientific, LLC. The other authors declare that the research was conducted in the absence of any commercial or financial relationships that could be construed as a potential conflict of interest.

## Supplementary Material

FIGURE S1 Inner phalangeal and border cells labeling by *Plp-CreER^T2^::Rosa26^tdTomato^*. **(A–C)** Representative confocal images of *Plp-CreER^T2^::Rosa26^tdTomato^* samples injected with tamoxifen (Tam) at P0. tdTomato labeling allowed the measurement of the inner phalangeal and border cell region for quantification of Hes5-LacZ and Sox2 expressing cells in **Figure 2**. This width was 9.6 μm at P2, 13.2 μm at P4, and 16.8 μm at P6. Scale bar = 25 μm.

FIGURE S2 Expansion of the Sox2-postive region and HC disorganization when *N1ICD* is overexpressed. **(A–D)** Representative confocal images taken from the apical turn of the cochlea from *Sox10^rtTA^::TetO-LacZ::TetO-N1ICD* mice that were injected with tamoxifen (Tam) at P0/P1 as well as administered doxycycline (Dox) in the diet to nursing mother with a doxycycline injection given to pups at P1 to induce expression of *N1ICD* and the *LacZ* reporter in SCs and other non-HCs. Cochlea were collected at P7 and stained with antibodies against myosin VIIa (red), Sox2 (white), and β-gal (green). While there was disorganization of HCs **(A)**, there was no change in the number of HCs in any of the cochlear turns. (**E**, quantified in a 200 μm region). Scale bar = 25 μm. *N* = 4. **(F–I)** Maximum projection images of *Sox10^rtTA^::TetO-LacZ* and **(J–M)** *Atoh1-DTA::Sox10^rtTA^::TetO-LacZ::TetO-N1ICD* apical turns showing expanded Sox2 expression medial and lateral to the organ of Corti. Scale bar = 20 μm.
