# Supplementary figures and images for: Spontaneous Hair Cell Regeneration Is Prevented by Increased Notch Signaling in Supporting Cells
Source: Front Cell Neurosci. 2018 May 4;12:120. doi: 10.3389/fncel.2018.00120 (PMC5945818; doi:10.3389/fncel.2018.00120)

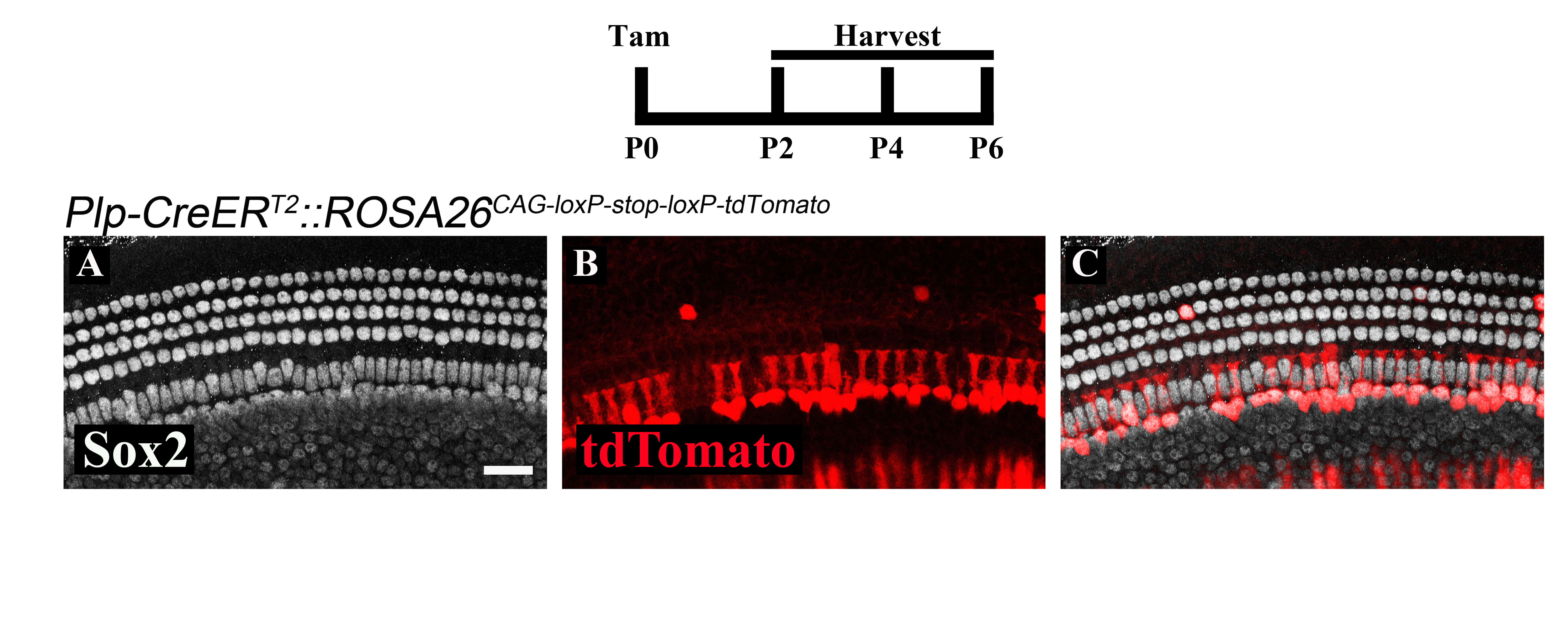

Supplement: FIGURE S1 — Inner phalangeal and border cells labeling by Plp-CreERT2::Rosa26tdTomato. (A–C) Representative confocal images of Plp-CreERT2::Rosa26tdTomato samples injected with tamoxifen (Tam) at P0. tdTomato labeling allowed the measurement of the inner phalangeal and border cell region for quantification of Hes5-LacZ and Sox2 expressing cells in Figure 2. This width was 9.6 μm at P2, 13.2 μm at P4, and 16.8 μm at P6. Scale bar = 25 μm. [file Image_1.TIF]

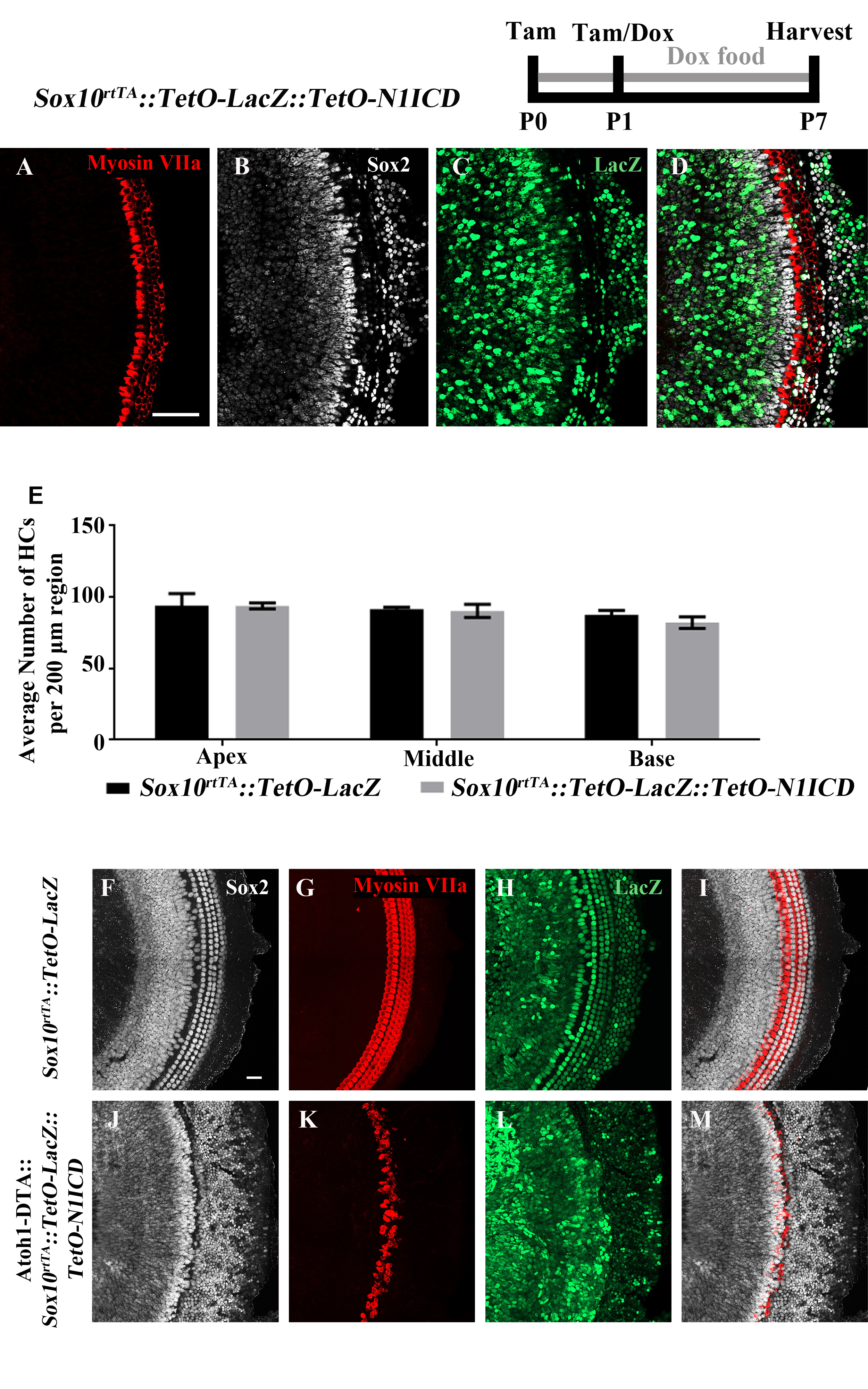

Supplement: FIGURE S2 — Expansion of the Sox2-postive region and HC disorganization when N1ICD is overexpressed. (A–D) Representative confocal images taken from the apical turn of the cochlea from Sox10rtTA::TetO-LacZ::TetO-N1ICD mice that were injected with tamoxifen (Tam) at P0/P1 as well as administered doxycycline (Dox) in the diet to nursing mother with a doxycycline injection given to pups at P1 to induce expression of N1ICD and the LacZ reporter in SCs and other non-HCs. Cochlea were collected at P7 and stained with antibodies against myosin VIIa (red), Sox2 (white), and β-gal (green). While there was disorganization of HCs (A), there was no change in the number of HCs in any of the cochlear turns. (E, quantified in a 200 μm region). Scale bar = 25 μm. N = 4. (F–I) Maximum projection images of Sox10rtTA::TetO-LacZ and (J–M) Atoh1-DTA::Sox10rtTA::TetO-LacZ::TetO-N1ICD apical turns showing expanded Sox2 expression medial and lateral to the organ of Corti. Scale bar = 20 μm. [file Image_2.tif]
